# Supplementary material for: Reconciling Mining with the Conservation of Cave Biodiversity: A Quantitative Baseline to Help Establish Conservation Priorities
Source: PLoS One. 2016 Dec 20;11(12):e0168348. doi: 10.1371/journal.pone.0168348 (PMC5173368; doi:10.1371/journal.pone.0168348)
Supplement: S1 Dataset — (ZIP) [file pone.0168348.s002.zip › Taxa/Serra Norte/SN_2013/N4E/Lista N4E-04.pdf]

CAVIDADE N4E-0004

Classe	Ordem	Fam/Outros	Gên/Outros	Espécie	S-U	U-S	Zon.
Annelida	Oligochaeta			jovem		1	P
Arachnida	Amblypygi	Phrynidae	*Heterophrynus*	*longicornis*	X		
Arachnida	Araneae	Araneidae	*Alpaida*	sp.1	X	X	E/P
Arachnida	Araneae	Ctenidae		jovem		1	E
Arachnida	Araneae	Filistatidae		sp.1	X		
Arachnida	Araneae	Ochyroceratidae	*Speocera*	sp.1		X	E/P
Arachnida	Araneae	Ochyroceratidae	*Theotima*	sp.	X	X	P
Arachnida	Araneae	Pholcidae	*Mesabolivar*	sp.	X		
Arachnida	Araneae	Pholcidae	*Metagonia*	sp.	X		
Arachnida	Araneae	Pholcidae		jovem	X	X	P
Arachnida	Araneae	Salticidae		sp.	X		
Arachnida	Araneae	Theridiidae	*Theridion*	sp.	X		
Arachnida	Araneae	Theridiosomatidae	*Plato*	sp.1	X	X	P
Arachnida	Araneae	Uloboridae		sp.	X		
Arachnida	Opiliones	Sclerosomatidae	Gagrellinae	sp.	X		
Arachnida	Opiliones	Stygnidae	*Protimesius*	*gracilis*	X		
Arachnida	Pseudoscorpiones	Chernetidae	*Spelaeochernes*	sp.1		X	E/P
Chilopoda	Scolopendromorpha	Cryptopidae	*Cryptops*	sp.3	X		
Chilopoda	Scutigeromorpha	Pselliodidae	*Sphendononema*	*guildingii*	X	1	E
Diplopoda	Polydesmida	Chelodesmidae		jovem	X		
Diplopoda	Polydesmida	Pyrgodesmidae		jovem	X	X	E
Entognatha	Collembola	Isotomidae		sp.1		X	P
Entognatha	Collembola	Paronellidae		sp.1		X	E/P
Entognatha	Collembola	Paronellidae		sp.3		X	E
Entognatha	Collembola			sp.	X		
Entognatha	Diplura	Campodeidae		sp.1	X	X	P
Insecta	Blattodea	Blattellidae		jovem	X	3	P
Insecta	Coleoptera	Carabidae		jovem	X		
Insecta	Coleoptera	Curculionidae	Scolytinae	sp.	X		
Insecta	Coleoptera	Lampyridae		jovem	X		
Insecta	Coleoptera	Leiodidae		sp.2		X	P
Insecta	Coleoptera	Nitidulidae		sp.	X		
Insecta	Diptera	Cecidomyiidae		sp.	X		
Insecta	Diptera	Chironomidae		sp.	X		
Insecta	Diptera	Mycetophilidae		sp.	X		
Insecta	Diptera	Phoridae	Phorinae	sp.		X	E
Insecta	Diptera	Psychodidae	Phlebotominae	sp.		X	E/P
Insecta	Heteroptera	Cydnidae		sp.	X	X	
Insecta	Heteroptera	Reduviidae	Reduviinae	sp.		2	P
Insecta	Homoptera	Cixiidae		jovem	X	X	P
Insecta	Hymenoptera	Braconidae		sp.	X		
Insecta	Hymenoptera	Formicidae	*Apterostigma*	sp.	X		
Insecta	Hymenoptera	Formicidae	*Camponotus*	*atriceps*	X		
Insecta	Hymenoptera	Formicidae	*Camponotus*	sp.1		X	P
Insecta	Hymenoptera	Formicidae	*Dolichoderus*	aff. *bispinosus*	X		
Insecta	Hymenoptera	Formicidae	*Gnamptogenys*	sp.1		X	P
Insecta	Hymenoptera	Formicidae	*Pachycondyla*	*constricta*	X		
Insecta	Hymenoptera	Formicidae	*Pheidole*	sp.2		X	P
Insecta	Lepidoptera	Tineoidea		sp.	X		
Insecta	Lepidoptera			jovem		X	P
Insecta	Orthoptera	Phalangopsidae	*Paraclodes*	sp.	X		
Insecta	Psocoptera			jovem	X	X	E/P
Gastropoda	Pulmonata	Subulinidae	*Lamellaxis*	sp.	X		
Gastropoda	Pulmonata	Subulinidae	*Obeliscus*	sp.	X		
Gastropoda	Pulmonata	Systrophiidae	*Happia*	sp.	X	X	P

Malacostraca	Isopoda	Armadillidae		sp.1		X	P
Malacostraca	Isopoda	Philosciidae		sp.1		X	P
Nematoda				sp.1		X	E
Mammalia	Chiroptera	Emballonuridae	*Peropteryx*	*kappleri*	5	6	P
Mammalia	Chiroptera	Phyllostomidae	*Micronycteris*	*microtis*		4	E
